# GlyCAM1 negatively regulates monocyte entry into the optic nerve head and contributes to radiation-based protection in glaucoma

**DOI:** 10.1186/s12974-017-0868-8

**Published:** 2017-04-26

**Authors:** Pete A. Williams, Catherine E. Braine, Nicole E. Foxworth, Kelly E. Cochran, Simon W. M. John

**Affiliations:** 1 The Jackson Laboratory, Bar Harbor, ME USA; 2 Department of Ophthalmology, Tufts University of Medicine, Boston, MA USA; 3 The Howard Hughes Medical Institute, Bar Harbor, ME USA

**Keywords:** Glaucoma, GlyCAM1, Monocytes, Retinal ganglion cell, Radiation therapy

## Abstract

**Background:**

We previously reported a profound long-term neuroprotection subsequent to a single radiation-therapy in the DBA/2J mouse model of glaucoma. This neuroprotection prevents entry of monocyte-like immune cells into the optic nerve head during glaucoma. Gene expression studies in radiation-treated mice implicated *Glycam1* in this protection. *Glycam1* encodes a proteoglycan ligand for L-selectin and is an excellent candidate to modulate immune cell entry into the eye. Here, we experimentally test the hypothesis that radiation-induced over-expression of *Glycam1* is a key component of the neuroprotection.

**Methods:**

We generated a null allele of *Glycam1* on a DBA/2J background. Gene and protein expression of *Glycam1*, monocyte entry into the optic nerve head, retinal ganglion cell death, and axon loss in the optic nerve were assessed.

**Results:**

Radiation therapy potently inhibits monocyte entry into the optic nerve head and prevents retinal ganglion cell death and axon loss. DBA/2J mice carrying a null allele of *Glycam1* show increased monocyte entry and increased retinal ganglion cell death and axon loss following radiation therapy, but the majority of optic nerves were still protected by radiation therapy.

**Conclusions:**

Although GlyCAM1 is an L-selectin ligand, its roles in immunity are not yet fully defined. The current study demonstrates a partial role for GlyCAM1 in radiation-mediated protection. Furthermore, our results clearly show that GlyCAM1 levels modulate immune cell entry from the vasculature into neural tissues. As *Glycam1* deficiency has a more profound effect on cell entry than on neurodegeneration, further experiments are needed to precisely define the role of monocyte entry in DBA/2J glaucoma. Nevertheless, GlyCAM1’s function as a negative regulator of extravasation may lead to novel therapeutic strategies for an array of common conditions involving inflammation.

**Electronic supplementary material:**

The online version of this article (doi:10.1186/s12974-017-0868-8) contains supplementary material, which is available to authorized users.

## Background

Glaucoma is a complex, multifactorial disease characterized by the progressive dysfunction and loss of retinal ganglion cells. It is a leading cause of vision loss affecting approximately 80 million people worldwide [1]. The earliest neurodegenerative events that injure retinal ganglion cell axons during glaucoma are unknown but multiple lines of inquiry have suggested a complex neuroinflammatory process involving multiple cell types [2–12].

The DBA/2J (D2) mouse is widely used as a model of age-related chronic glaucoma as it shares hallmark features with the human disease. In D2 mice, by 12 months of age, glaucomatous damage is present in the majority of eyes in our colony [13]. D2 glaucoma is characterized by a loss of retinal ganglion cells and axonal degeneration in the optic nerve. This axonal damage likely initiates at the optic nerve head (ONH) where the axons are insulted [14]. Early during D2 glaucoma, a class of pro-inflammatory monocyte-like cells exit the vasculature and enter the ONH [5]. Current experiments support a damaging role for these monocyte-like cells in early retinal ganglion cell axon injury [5]. Our lab has previously shown that this cell entry can be prevented by a single, sub-lethal dose of γ-radiation or by a local (eye-only) dose of X-ray radiation [5, 15]. This treatment subsequently provides life-long protection against glaucoma and has been confirmed by others [16]. The protection is profound with up to 96% of eyes developing no glaucoma [5, 15].

In order to mechanistically understand this radiation-based protection, Howell et al. performed gene expression profiling on optic nerve heads of pre-diseased and diseased mice. They compared expression in retinas and optic nerve heads from untreated D2 mice, strain-matched control D2-*Gpnmb* ^+^ mice that do not develop elevated intraocular pressure (IOP) or glaucoma, and γ-radiated mice at different ages post-radiation therapy [5, 6]. These data identified four genes that were differentially expressed following radiation therapy at a young age (4 months of age) and continued to be differentially expressed at ages up to 12 months of age (*Gfap*, *Glycam1*, *Npr3*, and *Stat3*) [5, 6]. Of these, increased *Glycam1* expression was consistent between the retina and the tissue punch of the optic nerve head (that included adjacent choroidal tissue), making GlyCAM1 an excellent candidate to participate in the radiation-induced protection.

GlyCAM1 (*Gly*cosylation-dependent *c*ell *a*dhesion *m*olecule *1*) is a proteoglycan ligand for L-selectin, modulating transendothelial migration of leukocytes [17–21]. GlyCAM1 is an unusual L-selectin ligand in that it lacks a transmembrane domain and acts as a peripheral membrane-associated protein on the vascular endothelium [18, 19]. GlyCAM1 is also secreted into the blood and may be a regulatory molecule that can directly bind to free leukocytes in the blood (as may other soluble L-selectin ligands) [17, 22–25]. Existing evidence supports a role for GlyCAM1 as a negative regulator of extravasation. GlyCAM1 levels (protein and mRNA) decrease during acute antigen-primed inflammation and depletion of soluble L-selectin ligands (using L-selectin-IgG affinity columns) decreases the inhibitory effect of mouse plasma on leukocyte binding to endothelial cells of high endothelial venules in Stamper-Woodruff assays [17, 23, 26]. In addition, selectin ligands that carry sialyl Lewis^X^ glycans, as does GlyCAM1, inhibit leukocyte binding to lymph nodes in an in vitro assay [21]. *Glycam1* is stably up-regulated following radiation treatment in glaucoma and monocyte entry into the optic nerve head is inhibited [5]. This is consistent with a role for GlyCAM1 as a negative regulator of monocyte extravasation with the increased GlyCAM1 levels inhibiting extravasation in radiation-treated eyes. However, and to our knowledge, a definitive physiologic role for GlyCAM1 as a negative regulator of extravasation has not been experimentally demonstrated by directly and specifically removing *Glycam1* in vivo. Similarly, whether specifically increasing GlyCAM1 levels beyond those typical for non-inflamed tissues can negatively modulate extravasation is not reported (GlyCAM1 levels decrease in HEVs during acute inflammation). Here, we generated a D2 substrain lacking *Glycam1* to determine if GlyCAM1 is important for the radiation-induced neuroprotection and to determine if increased levels of GlyCAM1 impede extravasation in glaucoma.

## Methods

### Mouse strain, breeding, and husbandry

The mice were housed and fed in a 14-h light/10-h dark cycle with food and water available ad libitum. All breeding and experimental procedures were undertaken in accordance with the Association for Research for Vision and Ophthalmology Statement for the Use of Animals in Ophthalmic and Visual Research. The Institutional Biosafety Committee (IBC) and the Animal Care and Use Committee (ACUC) at The Jackson Laboratory approved this study. The DBA/2J (D2) strain was utilized and has been described in detail elsewhere [13]. D2.B6N-*Glycam1* ^tm1(KOMP)Vlcg^ (D2.*Glycam1* ^*−/−*^) mice were generated by backcrossing C57BL/6JN.*Glycam1* ^tm1(KOMP)Vlcg^ mice, heterozygous for the *Glycam1* ^tm1(KOMP)Vlcg^ allele, to DBA/2J a minimum of six times (>N6) before intercrossing to generate mice homozygous for the *Glycam1* ^tm1(KOMP)Vlcg^ allele (>N6F1). The presence of the allele was confirmed by standard PCR genotyping, and *Glycam1* levels were confirmed by quantitative RT-PCR. Equal numbers of male and female mice were used.

### γ-Radiation therapy

A sub-lethal dose of γ-radiation (7.5 Gy) was administered using a ^137^Cesium source in a single dose at 10–12 weeks of age. The mice were placed on a rotating platform to ensure uniform administration of the treatment. The mice were monitored follow radiation treatment. This level of treatment does not result in any adverse conditions, and the mice do not require bone marrow reconstitution [5, 15].

### Axon labelling with PPD and assessment of glaucomatous damage

The processing of optic nerves and staining with paraphenylenediamine (PPD) was as published [27]. PPD stains the myelin sheath of all axons but darkly stains the axoplasm of only damaged axons. It is well established to provide a very sensitive measure of optic nerve damage [5, 6, 13]. Briefly, intracranial portions of optic nerves were fixed in 4% PFA at RT for 48 h, processed, and embedded in plastic. A retro-orbital segment of optic nerve from within a region up to 1 mm from the posterior surface of the sclera was sectioned (1-μm-thick sections) and stained with PPD. Typically, 30–50 sections are taken from each nerve. Multiple sections of each nerve were considered when determining damage level. The optic nerve was analysed by at least two individuals blinded to genotype and determined to have one of three damage levels:

1. No or early damage (NOE)—less than 5% axons damaged and no gliosis. This level of damage is seen in age- and sex-matched non-glaucomatous mice and is not due to glaucoma. Although none of these eyes exhibit glaucomatous nerve damage, this damage level is called no or early glaucoma as some of these eyes have early molecular changes that precede neurodegeneration. These molecular changes can be detected by gene expression studies [6]. The eyes with these early molecular changes but no degeneration are considered to have early glaucoma when discussing changes to monocyte extravasation in this paper.
2. Moderate damage (MOD)—average of 30% axon loss and early gliosis
3. Severe damage (SEV)—>50% axonal loss and damage with prominent gliosis

### Flow cytometry

The mice were euthanized, and the eyes were enucleated and placed immediately into ice-cold HBSS. The optic nerve heads were dissected from the eyes in HBSS on ice and placed directly into a dispase and DNase solution. The samples were incubated for 30 min at 37 °C and shaken at 350 RPM. Following this, the samples were blocked in 2% BSA in HBSS and stained with antibodies against CD11b (PE), CD11c (FITC), and CD45.2 (APC). The samples were stained with propidium iodide (PI) for 5 min before being run on the flow cytometer. Four-colour flow cytometry was performed on a FACSCaliber (BD Bioscience), and the samples were run to completion. Cell populations were analysed using FloJo. For whole-blood leukocyte counts, the blood was collected from restrained mice via cheek bleed before being stained with PI and LDS751. The samples were run on a Guava flow cytometer (EMD-Millipore).

### Immunofluorescent staining of retinal whole mounts and sections

The mice were euthanized, and their eyes were enucleated and placed in 4% PFA overnight (ON). For immunofluorescent staining retinas for cell counts, the retinas were dissected and flatmounted onto slides, permeabilized with 0.1% Triton-X for 15 min, blocked with 2% BSA in PBS, and stained ON at room temperature (RT) in primary antibody (1:500 with PBS; rabbit anti-RBPMS; Novus; NBP2-20112). After primary antibody incubation, the retinas were washed five times in PBS, stained for 4 h at RT with secondary antibody (AF594, ThermoFisher). The slides were then washed a further five times with PBS, stained with DAPI for 15 min, mounted with fluoromount, and coverslipped and sealed with nail-polish. For retinal sections, the eyes were cryoprotected in 30% sucrose ON, frozen in OCT, and cryosectioned at 18 μm. The slides were warmed to room temperature and the procedure above was followed. For GlyCAM1 staining, crude rabbit serum was used 1:50 in PBS (crude serum was prepared in rabbits after immunization against the peptide P2 (CKEPSIFREELISKD) coupled to KLH). GlyCAM1 rabbit serum was a kind gift from Jean-Philippe Girard and Nathalie Ortega (Institut de Pharmacologie et de Biologie Structurale, France) and generated in the lab of Steve Rosen (UCSF). For MECA-79 staining, a conjugated anti-MECA-79 AF488 antibody (eBiosciences; 53-6036-80) was used. The retinas were imaged on a Zeiss Axio Observer for low resolution counts, and the counting paradigm outlined in [28] was followed. Retinal sections were imaged on a Leica SP8 for higher resolution images and processed using Imaris (Bitplane).

### Nissl staining of frozen retinal sections

The mice were euthanized, and their eyes were enucleated and placed in 4% PFA ON. Following this, the eyes were cryoprotected in 30% sucrose, frozen in OCT, and cryosectioned at 18 μm. The slides were warmed to RT, placed in 1:1 alcohol:chloroform ON, and rehydrated through serial alcohol gradient. The slides were washed once in distilled water and stained for 15 min in 0.1% cresyl violet in distilled water before being differentiated in 95% alcohol, dehydrated in 100% alcohol, and cleared in xylene. The slides were left to dry at RT, mounted with fluoromount, and coverslipped and sealed with nail-polish. The sections were imaged using a Nikon Eclipse E200.

### Clinical examination and intraocular pressure measurements

D2 mice develop elevated intraocular pressure and glaucoma subsequent to an iris disease [13, 29]. In all D2 glaucoma experiments, the progression of the iris disease and intraocular pressure in mutant or drug-treated mice were compared to control D2 mice using previously described methods [30, 31]. In each experiment, iris disease and intraocular pressure were assessed in ≥40 eyes per genotype or treatment. Iris disease was assessed at 2-month intervals starting at 6 months of age until experiment completion. Intraocular pressure was measured at 45-day intervals beginning at 8.5–9 months of age until experiment completion. Statistical analysis was performed using Student’s *t* tests.

### Quantitative RT-PCR

Total RNA (40 ng) from retinal or choroidal tissue was reverse transcribed using the MessageSensor Reverse Transcriptase kit (Ambion) with random decamer priming (Ambion) according to the manufacturer’s protocols. A portion of the cDNA was then used in a PCR reaction containing Taqman Univeral PCR Master Mix (Applied Biosystems). The gene-specific primers and probe sets were obtained from Applied Biosystems and used according to the manufacturer’s protocols (*Glycam1*; Mm00801716_m1). Real-Time PCR was performed on a ViiA 7 system (Applied Biosystems) with the standard protocol of 95 °C for 10 min to activate the DNA polymerase followed by 40 cycles of amplification. The threshold cycle (Ct) was determined using the QuantStudio Real-Time PCR software (version 1.2).

### Statistical analysis

The sample size (number of eyes, *n*) is shown in each figure legend. Graphing and statistical analysis was performed in R. Student’s *t* test was used for pairwise analysis in quantitative plots, and Fisher’s exact test was used for nerve grade comparisons. Error bars refer to standard error of the mean unless otherwise stated. *P < 0.05, **P < 0.01, and ***P < 0.001.

## Results

### Whole-blood leukocyte levels are restored following gamma radiation

**Fig. 1:**
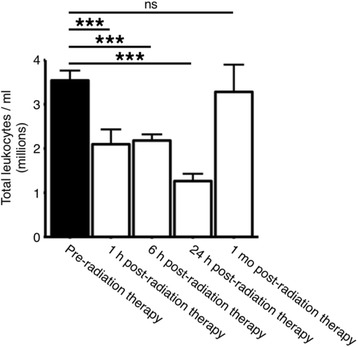
Whole-blood leukocyte levels return to normal levels following radiation therapy. Mice were γ-irradiated at 2–3 months of age, and whole-blood samples were taken directly before irradiation, 1 h, 6 h, 24 h, or 1 month following radiation therapy (*n* = 5 for all time-points). Whole-blood leukocyte numbers were depleted shortly after radiation therapy but returned to normal within 1 month. Thus, the inhibition of monocyte-like cell entry at the optic nerve head following radiation therapy is unlikely to be due to long-term depletion of leukocytes. ***P < 0.001. *ns* no significant difference (*P* > 0.05)

We have previously shown a protective effect for sub-lethal γ-radiation in the D2 model of glaucoma [15]. In this model, a single dose of 7.5 Gy γ-radiation at 2–3 months of age (which is 3–4 months before obvious iris disease and ~6 months before the onset of high intraocular pressure in most eyes in our colony) is able to protect from retinal ganglion cell loss and optic nerve degeneration. Importantly, the iris disease and IOP elevation are unchanged by the radiation therapy. Targeted X-ray irradiation of a single eye shows that the protective effects of radiation therapy are local to the eye [5], and so protective effects are unlikely to involve altered numbers of peripheral immune cells. To further confirm that peripheral immune cell numbers are grossly unchanged, we performed whole-blood leukocyte counts at differing time-points after a recovery period following radiation therapy (Fig. 1). As expected, blood leukocyte levels decreased shortly after radiation therapy (1–6 h) peaking at 24-h post-radiation therapy (total blood leukocyte numbers (cells/ml ± SEM); pre-radiation 3,540,007 ± 222,795; 1 h 2,096,131 ± 336,522; 6 h 2,180,040 ± 140,535; 24 h 1,263,976 ± 163,514). Cell levels were fully recovered to pre-radiation therapy levels within 1 month (3,280,827 ± 615,663) (*n* = 5/group). Given that radiation therapy is performed at a time-point far preceding clinical presentation of disease in our colony, depletion of peripheral blood leukocytes is unlikely to be a driver of the radiation-therapy-induced neuroprotection.

### *Glycam1* is up-regulated following radiation therapy and is localized to vasculature of the choroid and the vasculature surrounding the optic nerve head

In the eyes that have undergone radiation therapy, there is steady-state up-regulation of *Glycam1* expression, but how *Glycam1* influences radiation therapy and whether over-expression of *Glycam1* limits monocyte-like cell entry into the optic nerve head following radiation therapy is unknown.

**Fig. 2:**
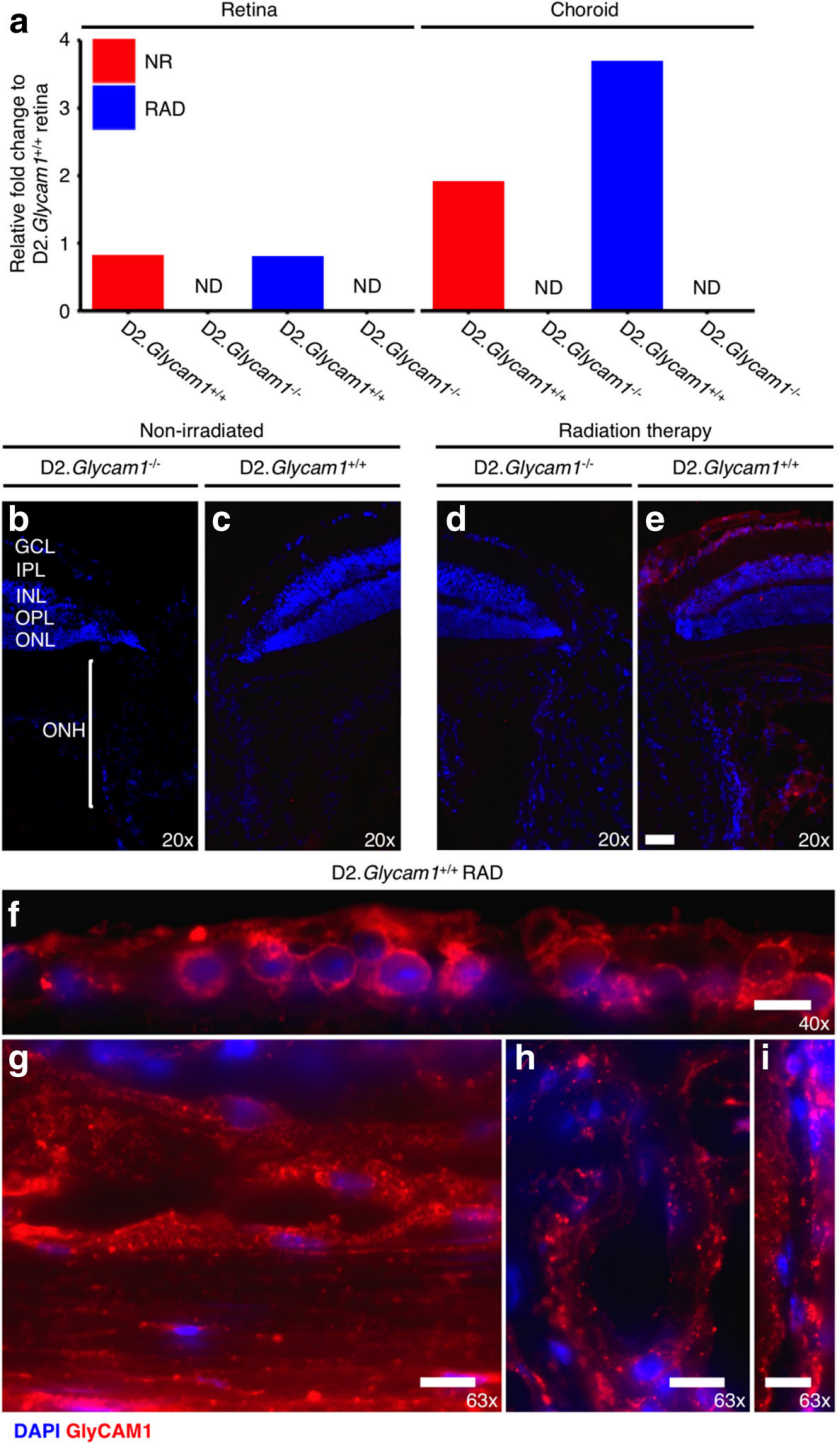
*Glycam1* gene and protein are up-regulated in the long term following radiation therapy. **a** *Glycam1* gene expression following radiation therapy. Retina and choroidal tissue were collected from D2.*Glycam1* ^−/−^ mice and wild-type counterparts (D2.*Glycam1* ^*+/+*^) at 12 months of age, and quantitative RT-PCR was performed. Untreated wild-type mice showed low (but present) levels of *Glycam1* in the retina and choroidal tissue. This was significantly increased in wild-type mice that had undergone radiation therapy, especially in the choroid. As expected, *Glycam1* was absent from D2.*Glycam1* ^−/−^ eyes (*n* = 8/group). **b**–**g** GlyCAM1 protein expression following radiation therapy. Immunofluorescence staining of GlyCAM1 (*red*) shows no expression in non-irradiated D2 mice or D2.*Glycam1* ^−/−^ eyes or irradiated D2.*Glycam1* ^−/−^ eyes (**b**–**d**). However, there is robust staining of GlyCAM1 within the ONH vasculature and choroidal vasculature following radiation therapy (overview in **e**, choroid **g**, ONH vasculature **h**, **i**). There is also GlyCAM1 staining in the inner retina directly on an around cells in the ganglion cell layer (**f**). This expression is long-lived (tissue was sampled at 12 months). Numbers in lower corners show relative magnification. Scale bars = 50 μm (**b**–**e**), 10 μm (**f**–**i**), *n* = 6/group

**Fig. 3:**
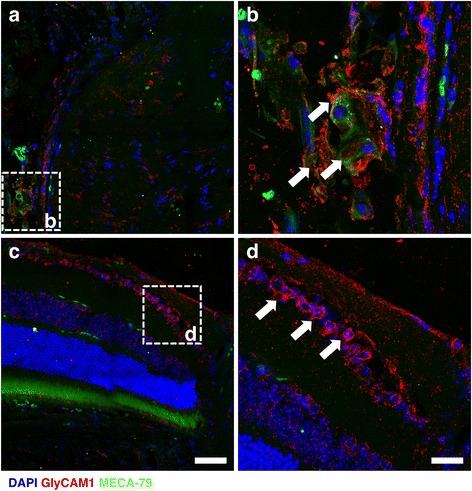
GlyCAM1 in the ONH is sulfated whereas GlyCAM1 in the inner retina is un-sulfated. We tested tissue for binding of MECA-79, an antibody which is dependent on sulfation to bind to GlyCAM1. Co-localization of MECA-79 (*green*) and GlyCAM1 (*red*) determined that the GlyCAM1 present in the vasculature local to the optic nerve is sulfated (*white arrows*, **a**, **b**) and that the GlyCAM1 present in the inner retina and within capillaries within the optic nerve is un-sulfated (*white arrows*, **c** and inset **d**). Scale bars = 50 μm (**a**, **c**), 20 μm (**b**, **d**), *n* = 4/group

To investigate the role of *Glycam1* in radiation-therapy in glaucoma, we developed the D2 mice with a null allele of *Glycam1* (D2.*Glycam1* ^*−/−*^). We then determined the expression pattern of *Glycam1* in untreated (non-irradiated, NR) and radiation-treated eyes of wild-type (D2.*Glycam1* ^*+/+*^) mice (Fig. 2). Quantitative RT-PCR on retinal and choroidal segments of tissue detected very low *Glycam1* expression in the NR mice but vastly increased expression in the radiation-treated mice, especially in vasculature of the choroid and vasculature surrounding the optic nerve head (*n* = 8/group) (Fig. 2a). There was no detectable *Glycam1* in NR or radiation-treated ONH that lacked the surrounding vasculature (as it was dissected off) by RT-PCR, (data not shown). Similarly, immunofluorescence of GlyCAM1 in frozen retina and optic nerve head sections detected no GlyCAM1 protein in the NR mice but robust staining following radiation treatment (present throughout the most inner retina, the accessory vasculature surrounding the ONH, and throughout the choroid) (*n* = 6/group). Surprisingly, GlyCAM1 protein expression was present not just within the vasculature but also directly on retinal ganglion cells (Fig. 2b–g). Subsequent staining with MECA-79 (which is dependent on sulfation to bind to GlyCAM1) determined that the GlyCAM1 present in the vasculature is sulfated and that the GlyCAM1 present in the inner retina is un-sulfated (*n* = 4/group) (Fig. 3). GlyCAM1 protein exists as both a bound and soluble form [22, 25, 32, 33]. Given that retinal ganglion cells do not express *Glycam1* and un-sulfated forms of GlyCAM1 are secreted from other tissues [34, 35], this un-sulfated GlyCAM1 may have been a soluble form secreted by vascular endothelial cells local to the retina and optic nerve head and then deposited in the inner retina. As there is increased vascular permeability in human glaucoma patients [36–38], it is possible that secreted soluble GlyCAM1 arrives at the inner retina through vascular leakage. It is also important to note that the increased *Glycam1* gene expression persists in the long term following radiation therapy (at least to 12 months, the oldest age assessed). Gene and protein expression of GlyCAM1 was not present in the D2.*Glycam1* ^*−/−*^ mice.

### *Glycam1* prevents monocyte-like cell entry into the optic nerve head during radiation therapy

**Fig. 4:**
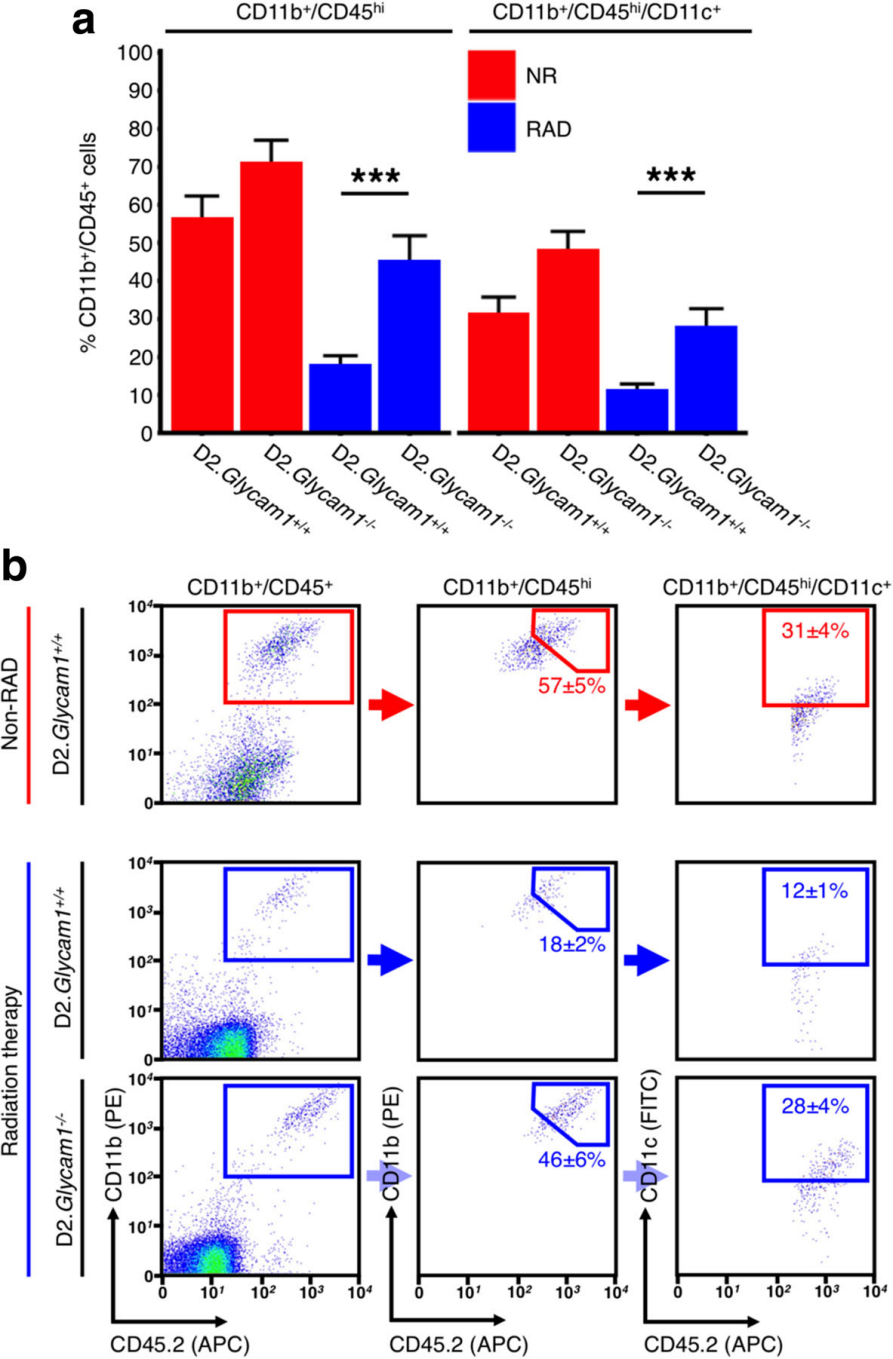
GlyCAM1 prevents inflammatory monocyte entry following radiation therapy. ONHs were collected from 12-month D2.*Glycam1* ^−/−^ and wild-type control mice, cells dissociated, and flow cytometry performed for the inflammatory monocyte markers seen in D2 glaucoma (CD11b, CD11c, CD45) (*n* > 20/group). Non-irradiated D2.*Glycam1* ^*−/−*^ mice showed normal monocyte-like cell entry during glaucoma; however, following radiation therapy, some cell entry still occurred in D2.*Glycam1* ^*−/−*^ optic nerve heads that was most pronounced in the CD11b^+^/CD45^hi^ cell populations (**a**, *left*). There was no significant difference in CD11c^+^ cell populations (**a**, *right*). Flow plots are shown in **b**. Viable cells were gated based on fluorescent staining of CD11b and CD45 (*left* column), from which CD11b^+^/CD45^hi^ cells were gated (*centre* column), and subsequently CD11b^+^/CD45^hi^/CD11c^+^ cells. Average values ± standard error of the mean is shown within panels, ***P < 0.001. Thus, *Glycam1* has a role in determining monocyte-like cell entry following radiation therapy in D2 glaucoma

We next utilized the D2.*Glycam1* ^*−/−*^ mice to determine functional roles of GlyCAM1 in modulating extravasation and in the radiation-induced neuroprotection. The D2.*Glycam1* ^*−/−*^ mice were housed with littermate, wild-type controls and underwent the same clinical phenotyping as controls. We found the D2.*Glycam1* ^*−/−*^ mice to be grossly normal and fertile and to develop a typical D2 glaucoma (Additional file 1: Figure S1). In addition, the eyes from the D2.*Glycam1* ^*−/−*^ mice have normal retinal ganglion cell numbers ruling out major developmental abnormalities (Additional file 2: Figure S2). The D2.*Glycam1* ^*−/−*^ mice and wild-type controls were γ-irradiated at 2–3 months of age and aged to the two key glaucoma time-points of 10.5 and 12 months of age. Following this, the eyes and nerves were harvested and the optic nerve heads and retinas were dissociated for flow cytometry using key markers of monocyte-like cells previously implicated in glaucoma (CD11b^+^, CD45^hi^, and CD11c^+^). CD45^hi^ is a well-established marker for bone marrow-derived cells and is useful for distinguishing cells that have entered tissues from resident cells [5, 39, 40]. Cells with these marker profiles enter the retina and optic nerve heads of glaucomatous D2 eyes but do not increase in radiation-treated eyes, with a very limited number of CD11b^+^/CD45^hi^/CD11c^+^ cells being present in radiation-treated eyes [5]. The non-irradiated D2.*Glycam1* ^*−/−*^ mice had monocyte-like cell entry typical of D2 glaucoma (i.e., comparable to wild-type controls). However, *Glycam1* genotype profoundly altered cellular entry following radiation treatment with much greater entry in the D2.*Glycam1* ^*−/−*^ mice (% CD11b^+^/CD45^+^ that are CD11b^+^/CD45^hi^/CD11c^+^ ± SEM; NR D2.*Glycam1* ^*+/+*^ 31.8 ± 3.9, D2.*Glycam1* ^*−/−*^ 48.3 ± 4.7, RAD D2.*Glycam1* ^*+/+*^ 11.7 ± 1.2, D2.*Glycam1* ^*−/−*^ 28.3 ± 4.4) (*n* > 20/group) (Fig. 4). These data clearly demonstrate that *Glycam1* has a critical role in reducing monocyte-like cell entry into the ONH in DBA/2J glaucoma following radiation treatment. It is important to note that although blood leukocyte populations are depleted immediately following radiation therapy (<1 h), they recover again within 1 month (see above); thus, the inhibition of monocyte-like cell entry at the optic nerve head is unlikely to be due to long-term depletion of leukocytes.

### DBA/2J mice deficient in *Glycam1* are more vulnerable to retinal ganglion cell degeneration following radiation therapy

**Fig. 5:**
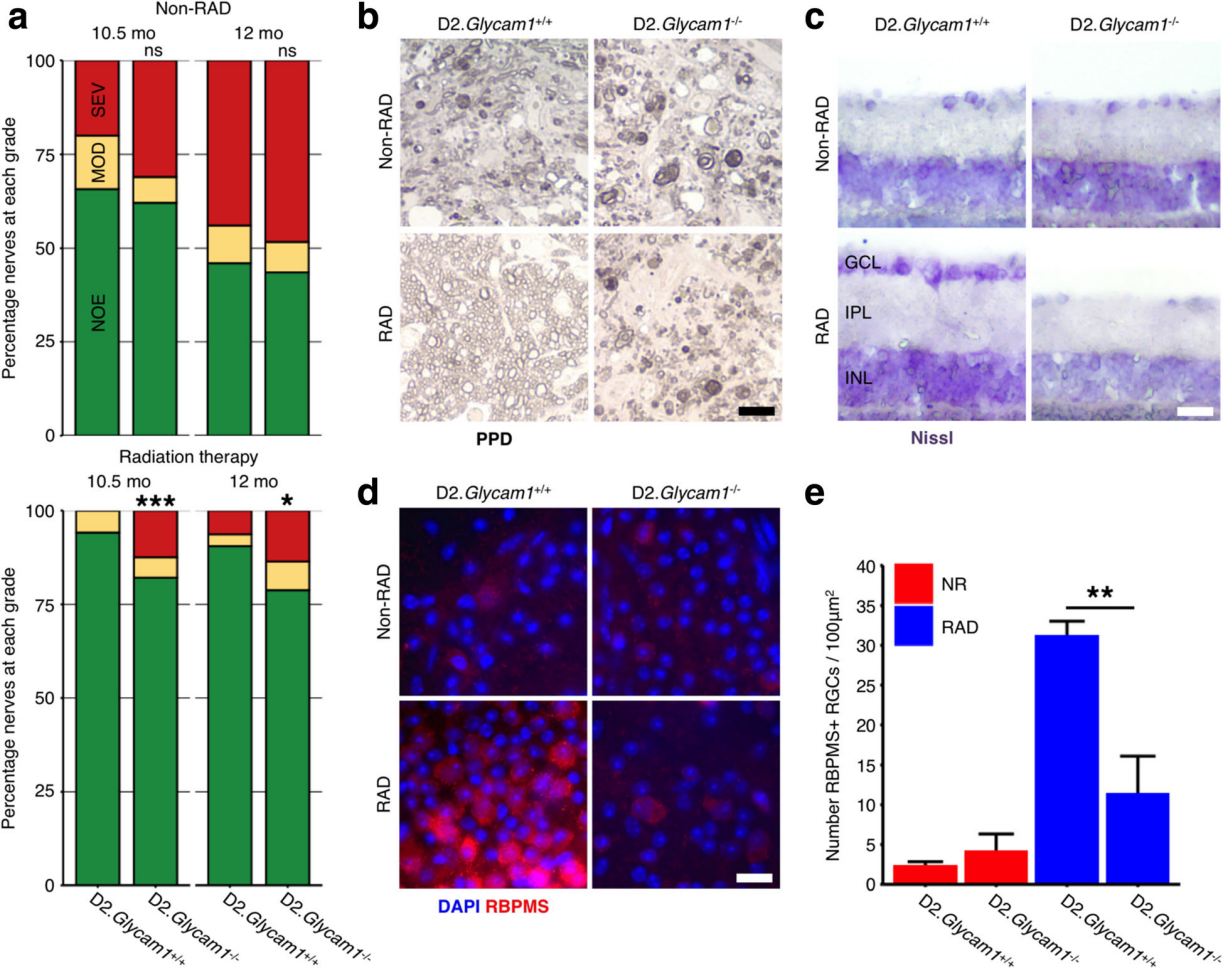
*Glycam1* is partially responsible for radiation therapy in glaucoma. Optic nerves are robustly protected in wild-type D2 eyes that have undergone radiation-therapy (RAD); however, this protection is less in mice carrying a null allele of *Glycam1*. D2.*Glycam1* ^−/−^ mice show significantly increased levels of optic nerve degeneration (as assessed by a sensitive PPD stain, **a** and examples in **b**) following radiation therapy (*n* > 50/group). This was most pronounced for the 10.5-month time-point. Since somal and axon degeneration can occur independently, we determined if retinal ganglion cells from D2.*Glycam1* ^−/−^ mice had degenerated within the retina (as opposed to just axon segments within the optic nerve having degenerated). Retinas were sectioned and Nissl stained (*n* = 4/group) (**c**) or flatmounted and stained with an antibody against RBPMS (a specific marker of retinal ganglion cells) and with DAPI to counterstain all cells within the inner retina (including amacrine cells, astrocytes, and microglia, as well as retinal ganglion cells [69]) (*n* = 8 representative counted regions per retina (as in [28]) from 6 eyes/group) (**d**, **e**). In wild-type, untreated mice, there is widespread loss of retinal ganglion cells at 12 months of age, and this was prevented in radiation-treated controls. Non-irradiated D2.*Glycam1* ^*−/−*^ mice had a similar pattern and degree of retinal ganglion cell loss as wild-type controls. The protective benefit of radiation treatment was diminished in D2.*Glycam1* ^*−/−*^ mice with more mice developing severe optic nerve damage. (Example retinas from eyes with severe optic nerve damage are shown in correspondence to wild-type radiation-treated controls (bottom left panels in **b**–**d**).) Thus, the radiation-induced protection from neural damage and the extravastation of monocytes in glaucoma is at least partially dependent on *Glycam1*. Importantly, non-irradiated (non-RAD) D2.*Glycam1* ^−/−^ mice do not have an increased incidence of glaucomatous neurodegeneration. Thus, *Glycam1*’s role in lessening glaucoma is context dependent on radiation therapy. Scale bars; RBPMS = 20 μm (**a**), Nissl = 20 μm (**b**), PPD = 20 μm (**c**), **P < 0.01 (**d**), *P < 0.05, ***P < 0.001. *ns* non-significant (**a** Fisher’s exact test; **e** Student’s *t* test)

To explore whether radiation-based protection to the retina and optic nerve was also dependent on *Glycam1*, retinal ganglion cell number and optic nerve integrity were assessed in the non-irradiated and irradiated D2.*Glycam1* ^*−/−*^ mice (Fig. 5). To assess optic nerve damage, the optic nerves were harvested at 10.5 and 12 months and stained with PPD that labels the axoplasm of dead/dying axons (*n* > 50/group). The nerves were determined to have one of the three damage levels: “no or early” damage (NOE), “moderate” damage (MOD), or “severe” damage (SEV, see the “Methods” section). The non-irradiated D2.*Glycam1* ^*−/−*^ mice and wild-type controls had similar levels of optic nerve damage at each time-point (10.5 months *P* = 0.51, 12 months *P* = 0.88; Fisher’s exact test). The wild-type mice that underwent radiation therapy were robustly protected from optic nerve damage as previously reported. Although many eyes were still protected, a significantly greater portion of radiation-treated D2.*Glycam1* ^*−/−*^ eyes developed severe damage (*P* < 0.001 10.5 months, *P* < 0.05 12 months, Fisher’s exact test). Thus, *Glycam1* contributes to the neuroprotection but is not alone a major determinant. Given the complexity of D2 glaucoma, as well as the complex, context-dependent regulation of extravasation, it is not surprising that *Glycam1* does not solely mediate radiation-induced protection*.*

## Discussion

Glaucoma is a complex age-related disease. Although molecular mechanisms that drive glaucomatous neurodegeneration remain to be fully elucidated, our work and other’s have identified early axon damage within the optic nerve head, as well as other varied intrinsic and extrinsic factors, as early drivers of damage following periods of elevated IOP [4, 6, 14, 41–44]. An increasing body of literature suggests that neuroinflammation is likely to be a key process in glaucoma [42, 45–54]. The optic nerve head contains a putative blood-brain barrier controlling the passage of cells and molecules from the blood to the tissue local to the axons of retinal ganglion cells. The optic nerve head contains differing cell types (astrocytes, microglia, vascular endothelial cells, and pericytes) that all may play a role in regulating immune cell movement from the blood to the tissue local to the retinal ganglion cell axons. At this location, retinal ganglion cell axons remain mainly unmyelinated and thus may be particularly vulnerable to damage from immune-derived cells.

We previously discovered that monocyte-like cells enter the optic nerve head very early during DBA/2J glaucoma, and we proposed that the radiation-induced neuroprotection is at least partially mediated by preventing the extravasation of these cells [5]. Evidence for extravasation of these cells from vessels included cell labelling by CFDA within the spleen with subsequent homing of labelled cells to the optic nerve head and flow cytometry demonstrating that these cells had high expression of CD45 (CD45^hi^), a well-established marker for bone marrow-derived cells that circulate in the blood [5, 39, 40, 55]. The data did not support their derivation from resident cells within the eye; resident microglial cells have low levels of CD45 (CD45^lo^), and the number of CD45^lo^ cells was not detected to increase during glaucoma in these experiments [5]. The monocyte-like cells that enter the eye produce various molecules that are known to be damaging in glaucoma, including endothelin 2 and complement component C1 [3, 5]. The neuroprotective radiation treatment altered the activation of endothelial transmigration pathways and prevented the disease-dependent entry of these cells into the optic nerve head [5]. Adding endothelin 2, a damaging molecule that is made by these cells, induced a glaucoma-like phenotype in radiation-treated eyes that had high IOP, while combined treatment to inhibit the effects of two damaging mediators that are made by these cells (complement C1 and endothelin 2) was profoundly protective against glaucoma [5]. Together, these data argue for an important role of these infiltrated cells as damaging in DBA/2J glaucoma.

Others have confirmed the radiation-induced protection using DBA/2J mice [16], while another study of survivors of the Hiroshima and Nagasaki atomic bombs suggests that radiation exposure protects from human glaucoma [56]. However, when tested in an experimentally induced rat model of glaucoma, radiation was not found to be protective (however, there were many experimental differences and so the studies are not directly comparable) [57]. Thus, and although there is evidence for infiltrating immune cells and immune cell activation in multiple models of glaucoma [3, 5, 58–62], additional work is required to definitively establish their function and to extend these studies to other models of glaucoma and human glaucoma [54]. Examining the role of these cells and the intricacies of the molecular mechanisms by which radiation therapy inhibits cell entry may lead to important human therapeutics.

GlyCAM1 is a receptor for leukocytes binding to L-selectin (primarily expressed by endothelial cells) [24]. *Glycam1* is constitutively up-regulated following radiation therapy and may protect from glaucoma by negatively modulating extravasation. To definitively test this hypothesis, we used a complete knockout of *Glycam1* in D2 mice. We found that GlyCAM1 is an important negative modulator of extravasation and is partially responsible for radiation-based protection. To our knowledge, this is the first clear evidence of a physiological impact for increased GlyCAM1 in inhibiting extravasation in neural tissues. Despite the substantial impact of *Glycam1* deficiency on extravasation, its impact on the radiation-induced neuroprotection was small (although still significant). Thus, *Glycam1* contributes to the neuroprotection but is not alone a major determinant.

The profound effect of *Glycam1* deficiency on cell entry into the eye but small effect on radiation protection may argue that preventing cellular infiltration is not a substantial contributor to the neuroprotection. Along with our current demonstration of a partial role of *Glycam1* in mediating the neuroprotection, the presence of monocyte-like cells (at levels comparable to those in untreated wild-type eyes) in largely protected *Glycam1*-deficient irradiated eyes suggests that infiltrating cells are not the only contributors to glaucomatous damage. Alternatively or additionally, and although *Glycam1* deficiency overall promotes extravasation, its deficiency may largely prevent extravasation of a specific unidentified cell type that is highly potent in mediating glaucomatous damage. Another possibility is that the cells entering irradiated *Glycam1* deficient eyes do not have the same properties as those entering wild-type D2 eyes and that they are less capable of inducing glaucoma. This is a possibility as the molecular interactions during extravasation are an important determinant of the cell types that exit the vasculature and of their subsequent cellular phenotype [63–66]. Further experiments are needed to resolve the role of infiltrating cells in glaucoma. Important experiments will include evaluating the effects of *Glycam1* deficiency on the infiltrating cells (detailed characterization of their molecular phenotype) and evaluating the importance of other molecules that participate in extravasation in both glaucoma and the radiation-induced neuroprotection.

It is important to note that cell types other than infiltrating monocyte-like cells may be critical in the radiation protection [5, 15, 16]. Bosco and colleagues have reported increasing numbers of resident microglia in DBA/2J eyes at very early ages and show that early minocycline treatment protects from glaucoma (aimed at inhibiting damaging microglial activation) [11, 67]. In these experiments, they reported that the degree of early microgliosis correlates with the degree of glaucoma severity at older ages [11], that radiation treatment inhibits early microgliosis [16], and that inhibition of microgliosis may underlie the radiation-induced neuroprotection. On the other hand, and despite confirming microglial activation, our experiments have provided no evidence for increased numbers of resident microglia (CD11b^+^ CD45^lo^) at 10.5 months of age or differential activation of ramified microglia between radiation-treated and untreated eyes (using CD68 as a marker of activated microglia) [5]. Further, we have found that minocycline administration (started at older ages) actually made the glaucoma worse [68]. The reasons for these differences likely reflect substantial differences in the nature (assays) and timing (ages assessed and stage of disease when treatments were initiated) of experiments, as well as potential environmental differences between institutions, which can substantially modify the disease phenotype. Clearly, further experiments are needed to resolve the roles of resident microglia and infiltrating monocyte-like cells in glaucoma and the radiation-induced protection.

## Conclusions

In conclusion, we discover that transcription and protein expression of the proteoglycan ligand GlyCAM1 is increased in the long term following radiation therapy in D2 glaucoma. D2 mice carrying a null allele of *Glycam1* show increased monocyte entry to the ONH but only a small increase in the incidence of glaucomatous neurodegeneration following radiation therapy. Demonstrating a role for GlyCAM1 as a specific negative regulator of cell extravasation in neural tissue is important. These findings expand our understanding of the mechanisms of radiation therapy in glaucoma. Identifying GlyCAM1 as a molecule involved in radiation-based protection and as negative regulator of extravasation in neural tissue may lead to novel therapeutic strategies using GlyCAM1-mimetics to prevent extravasation at the vascular endothelium in an array of common diseases that involve inflammation and other neuroinflammations.

## Additional files

Additional file 1: Figure S1. D2.*Glycam1* ^−/−^ mice have typical D2 glaucoma disease progression. IOP profiles (a) and clinical presentation of glaucoma (b). Iris disease progressed at a similar rate and reached a severe state in all groups within the same time-frame. For boxplots, the upper and lower hinges represent the upper and lower quartiles. The centerline of each diamond (*red*) represents the mean, and the upper and lower diamond points represent 95% confidence intervals of the mean (*n* > 25 for all groups).

Additional file 2: Figure S2. D2.*Glycam1* ^−/−^ mice have normal retinal ganglion cell numbers. To rule out major developmental abnormalities in the D2.*Glycam1* ^−/−^ eyes, the eyes from 3 months D2.*Glycam1* ^−/−^ and age-matched wild-type controls (*n* = 4/group) were flatmounted and stained for RBPMS (a specific marker of retinal ganglion cells). There was no significant difference in retinal ganglion cell number between the D2.*Glycam1* ^−/−^ eyes and controls (*P* > 0.05, Student’s *t* test) (a). Examples shown in b. ns = non-significant, scale bar = 20 μm.

## Abbreviations

GCL: Ganglion cell layer
INL: Inner nuclear layer
IOP: Intraocular pressure
IPL: Inner plexiform layer
OCT: Optimal cutting temperature
ON: Overnight
RGC: Retinal ganglion cell
RT: Room temperature
