# Supplementary figures and images for: GlyCAM1 negatively regulates monocyte entry into the optic nerve head and contributes to radiation-based protection in glaucoma
Source: J Neuroinflammation. 2017 Apr 26;14:93. doi: 10.1186/s12974-017-0868-8 (PMC5406973; doi:10.1186/s12974-017-0868-8)

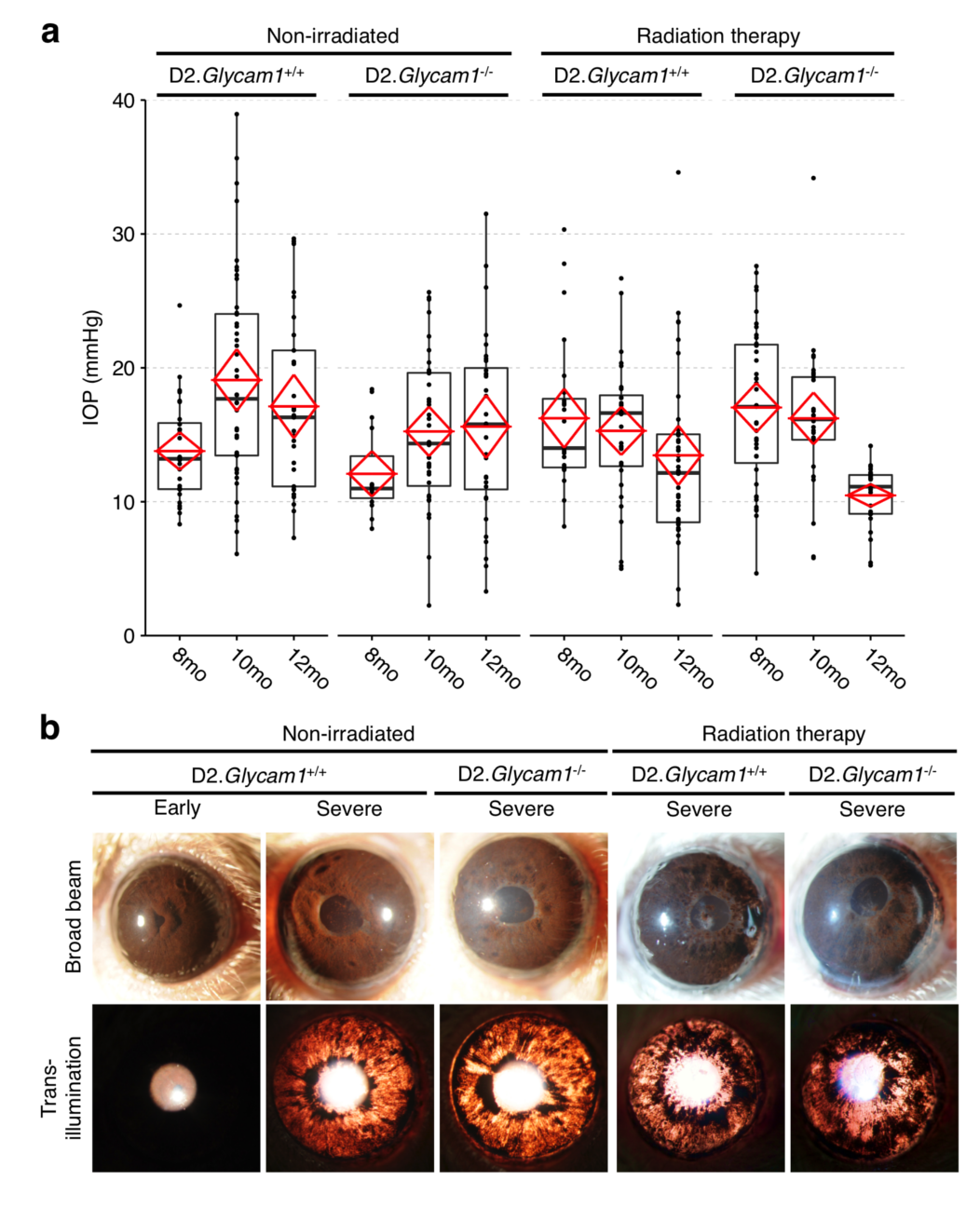

Supplement: Supplementary file 1 — D2.Glycam1 −/− mice have typical D2 glaucoma disease progression. IOP profiles (a) and clinical presentation of glaucoma (b). Iris disease progressed at a similar rate and reached a severe state in all groups within the same time-frame. For boxplots, the upper and lower hinges represent the upper and lower quartiles. The centerline of each diamond (red) represents the mean, and the upper and lower diamond points represent 95% confidence intervals of the mean (n > 25 for all groups). [file 12974_2017_868_MOESM1_ESM.tif]

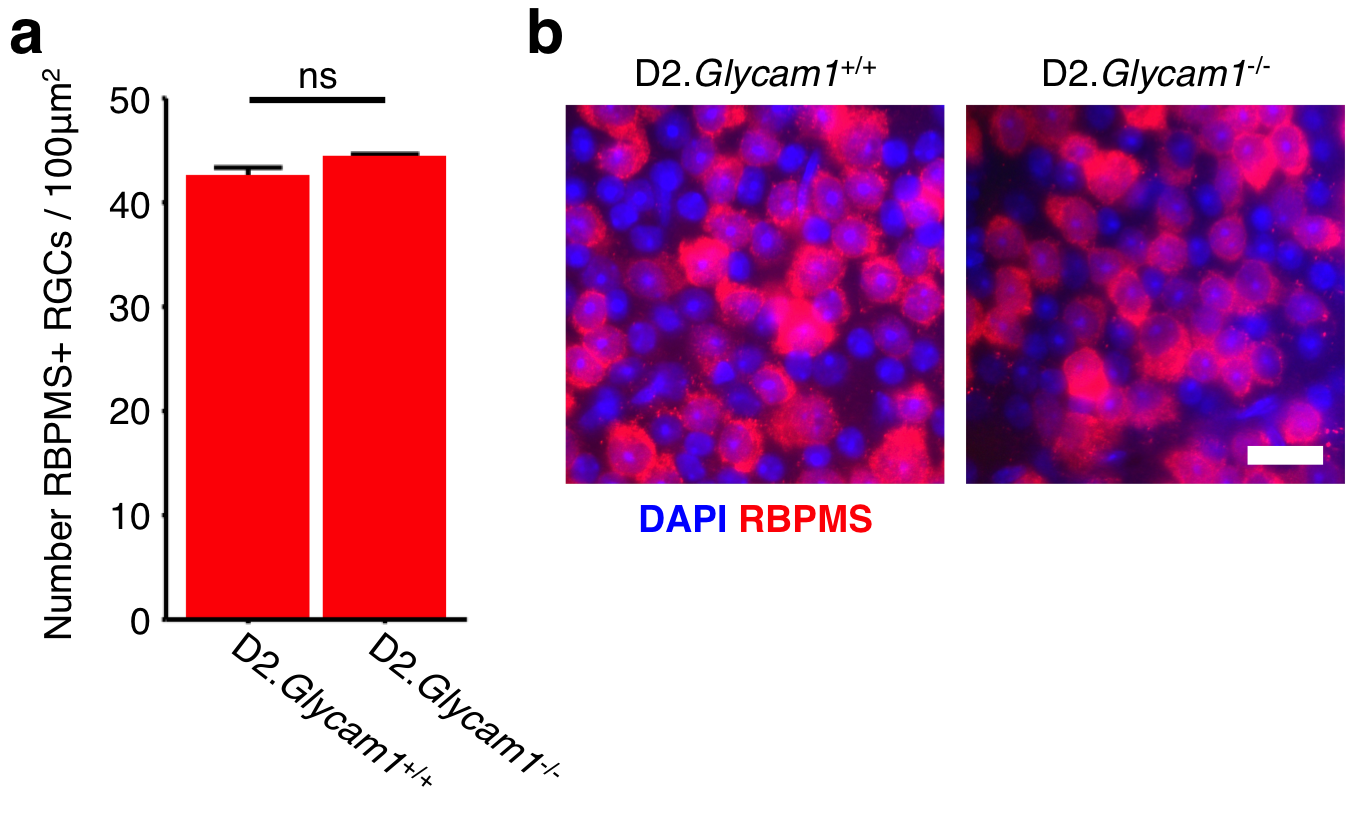

Supplement: Supplementary file 2 — D2.Glycam1 −/− mice have normal retinal ganglion cell numbers. To rule out major developmental abnormalities in the D2.Glycam1 −/− eyes, the eyes from 3 months D2.Glycam1 −/− and age-matched wild-type controls (n = 4/group) were flatmounted and stained for RBPMS (a specific marker of retinal ganglion cells). There was no significant difference in retinal ganglion cell number between the D2.Glycam1 −/− eyes and controls (P > 0.05, Student’s t test) (a). Examples shown in b. ns = non-significant, scale bar = 20 μm. [file 12974_2017_868_MOESM2_ESM.tif]
